# Efficacy of preoperative chemotherapy regimens in patients with initially unresectable locally advanced gastric adenocarcinoma: capecitabine and oxaliplatin (XELOX) or with epirubicin (EOX)

**DOI:** 10.18632/oncotarget.11818

**Published:** 2016-09-01

**Authors:** Yan Wang, Rong-yuan Zhuang, Yi-yi Yu, Shan Yu, Jun Hou, Yuan Ji, Yi-hong Sun, Kun-tang Shen, Zhen-bin Shen, Feng-lin Liu, Nai-qing Zhao, Tian-shu Liu

**Affiliations:** ^1^ Department of Medical Oncology, Zhongshan Hospital, Fudan University, Shanghai, China; ^2^ Department of Pathology, Fudan University, Zhongshan Hospital, Shanghai, China; ^3^ Department of General Surgery, Fudan University, Zhongshan Hospital, Shanghai, China; ^4^ Department of Biostatistics and Social Medicine, School of Public Health, Fudan University, Shanghai, China; ^5^ Center of Evidence-based Medicine, Fudan University, Shanghai, China

**Keywords:** locally advanced gastric cancer, preoperative chemotherapy, resection rate

## Abstract

**Purpose:**

We assessed the effectiveness of EOX (capecitabine, oxaliplatin and epirubicin) compared with XELOX (capecitabine and oxaliplatin) as preoperative chemotherapy for initially unresectable locally advanced gastric cancer.

**Methods:**

This is a prospective observational study. Patients with unresectable locally advanced gastric cancer were performed EOX regimen or XELOX regimen at the discretion of the investigators. They were assessed for response every 2 cycles by CT (computed tomography) scan. A multidisciplinary team reassessed resectability after 4 cycles. The primary endpoint was the response rate. Secondary end points included the R0 resection rate, survival and adverse events.

**Results:**

From November 2008 to May 2015, 242 patients were enrolled; 112 of them were assigned to EOX regimen and 130 to XELOX regimen. The response rates were 33.0% and 33.8% respectively in EOX group and XELOX group (*P* = 0.997). After 4 cycles of chemotherapy, 63 patients (56.3%) in EOX group and 81 patients (62.3%) in XELOX group received radical operation (*P* = 0.408). There was no significant difference in progress-free survival (PFS, 12.0m *vs*. 15.4m, *P* = 0.925) and overall survival (OS, 25.7m *vs*. 29.0m, *P* = 0.783) in two groups. In addition, more adverse effects occurred in EOX group, such as more leucopenia (22.3% *vs*. 10.0%, *P* = 0.014), neutropenia (23.2% *vs*. 11.5%, *P* = 0.025), fatigue (11.6% *vs*. 3.8%, *P* = 0.041) and vomiting (10.7% *vs*. 2.3%, *P* = 0.015).

**Conclusions:**

For unresectable locally advanced gastric cancer patients, XELOX regimen showed similar effects in response rate, radical resection rate and survival benefits, but with less toxicity effects.

## INTRODUCTION

Gastric cancer is one of the leading causes of cancer death worldwide [1]. Surgical resection is the curative treatment for the early stage gastric cancer [2]. However, gastric cancer patients are generally diagnosed at an advanced stage with extensive regional nodal involvement or invasion of adjacent structures. Only 50-60% of patients with newly diagnosed gastric cancer are suitable candidates for radical surgery with curative intent [3]. When radical surgery could not be done at first diagnosis, the prognosis of locally advanced gastric cancer (LAGA) patients is rather poor [4].

Some investigators have reported that initially unresectable locally advanced gastric cancer can be successfully treated with preoperative chemotherapy and followed radical resection [5–7]. However, the preoperative chemotherapy regimens were quite different in those trials. Epirubicin, cisplatin and continuous 5-fluorouracil (5-Fu) infusion (ECF) has been reported with high clinical response rate in advanced gastroesophageal adenocarcinoma and is currently the ‘gold standard’ chemotherapy regimen as preoperative chemotherapy in western countries [8]. Recently, the addition of docetaxel to cisplatin and fluorouracil in preoperative treatment were shown to improve the outcome of unresectable gastric cancer patients in Japan [9]. Although the response rates of these triplet combinations could be higher than the doublet combinations, severe toxicities like neutropenia and febrile neutropenia may limit their clinical practice. Meanwhile, it is a concerning issue whether patients could tolerate the same intensive regimen after operation.

In our experience, it has been demonstrated that doublet regimen such as XELOX regimen (capecitabine plus oxaliplatin) could produce favorable tumor response rate with relatively mild toxicity profile for advanced gastric cancer patients with para-aortic lymph node metastasis [10]. Therefore, since it is unknown whether more aggressive treatment with three chemotherapeutic agents produces better clinical effects compared to doublet therapy in enabling curative resection, the objective of this study is to determine what kind of chemotherapy strategy (EOX or XELOX regimen) can make subsequent radical surgery feasible and improve overall survival in patients with locally advanced gastric cancer.

## PATIENTS AND METHODS

### Patient selection

This is a prospective observational study. Patients with unresectable, histologically confirmed gastric or EGJ (Esophagogastric junction) adenocarcinoma with no distant metastases were eligible for the study from November 2008 to May 2015. All the patients received endoscopic examination, contrast CT scan for abdomen and pelvic, chest X-ray, as well as physical examination. Unresectability was judged by a local multidisciplinary team, according to one of the following criteria: radical resection was unable for technical reasons after laparotomy or laparoscopy exploration; tumor invades adjacent structures such as the pancreas, liver, diaphragm, adrenal gland or transvers colon (T4b) in CT scan; bulky lymph nodes (larger than 3 cm) along the celiac, splenic, common or proper hepatic arteries, or the superior mesenteric vein. Exclusion criteria of patients are: peritoneal metastasis confirmed by CT scan; lung metastasis, liver metastasis, pleural effusion, and/or other distant metastasis; serious uncontrolled comorbid conditions; any local intervention after initial diagnosis, such as surgical procedures, radiotherapy or TACE (trans-artery chemo-embolization); patients who could not comprehend or comply with the study. A multidisciplinary evaluation was required in this study. All patients signed an approved written informed consent. The protocol of this trial was approved by the institutional ethical board of Zhongshan Hospital, Fudan University and was registered on ClinicalTrials.gov (NCT02192983).

### Preoperative chemotherapy

All the patients received chemotherapy after successful enrollment. They were received EOX (group A) or XELOX (group B) regimen after physician's preference. EOX regimen was planned as capecitabine of 625 mg/m^2^, orally administered twice a day on days 1-14, oxaliplatin at 130 mg/m^2^ intravenous 2 h infusion and epirubicin at 50 mg/m^2^ on day 1. XELOX regimen was planned as capecitabine of 1000 mg/m^2^, orally administered twice a day on days 1-14 and oxaliplatin at 130 mg/m^2^on day 1, as intravenous 2 h infusion. Chemotherapy was repeated every three weeks.

### Tumor response and toxicity criteria

After every two cycles (6 weeks), an abdominal and pelvic CT scan was performed to evaluate the tumor response. Resectability was assessed by multidisciplinary team after four cycles of treatment. Resection was intended to be done within 4-6 weeks after the last treatment cycle. Patients with unresectable tumors continued treatment and were assessed for resectability every two cycles for a maximum duration of eight cycles or until progression. After resection, patients were continued the previous regimen for four cycles. Patients with progressive disease or unacceptable toxicity were treated at the discretion of the investigators. Response to the treatment was evaluated according to response evaluation criteria in solid tumor (RECIST) 1.1 [12] and for primary lesions according to the guidelines of the Japanese classification of gastric carcinoma [13]. The adverse events were assessed according to the Common Toxicity Criteria of the National Cancer Institute (NCI -CTC) 3.0 [14].

### Surgical procedure and pathological evaluation

The type of surgery performed depended on the location and extent of the primary cancer. The tumor was resected along with a gastric margin of more than 5 cm when feasible. For a distal tumor, a subtotal gastrectomy was considered, and total gastrectomy was performed for proximal cancers. An attempt was made to perform an extended LN resection (D2) in any patient who was qualified to have radical surgery. The surgical specimens were pathologically evaluated as grade0 when degeneration and/or necrosis were absent within the tumor, grade 1a when these areas accounted for less than one-third of the tumor, grade 1b when these areas accounted for more than one-third and less than two-thirds of the tumor, grade 2a when these areas accounted for more than two-thirds of the tumor, although tumor tissue apparently remained, grade 2b when only minimal tumor cells remained, and grade 3 when no residual tumor was detected. Pathological finding with grade 1b, 2a, 2b, or 3 were classified as pathological responders, while pathologic complete response (pCR) was defined as grade 3 [15].

### Post-operative treatment and follow-up

After R0 resection, adjuvant chemotherapy with the original regimen was initiated within 42 days of surgery, and eight cycles were administered during perioperative period. Patients who could not undergo a radical operation received palliative chemotherapy until evidence of disease progression appeared, and second-line treatment was recommended for adequate patients. All enrolled patients were followed up regularly. Physical and blood examinations were conducted every three months for the first three years and every six months thereafter. An abdominal CT scan was performed every six months for the first three years, and every year thereafter. Chest CT scan and upper gastrointestinal endoscopy were conducted every year.

### Statistical analysis

The primary study endpoint was the response rate, and secondary endpoints included R0 resection rate, progression-free survival (PFS), overall survival (OS) and toxicity. PFS was measured from the date of initial treatment to the first objective documentation of disease progression or relapse. OS was measured from the start of the treatment to the date of the last follow-up or death. All patients were followed up every three months.

Patient baseline characteristics and disease factors were summarized using descriptive statistics. The categorical parameters were compared using two-sided Pearson's test or Fisher's exact test, as appropriate. The PFS and OS were generated by the Kaplan-Meier method and were compared by means of the log-rank test. SPSS software (version 16.0; SPSS, Chicago, IL) was used for statistical analyses. A *P* < 0.05 was considered significant.

## RESULTS

### Baseline characteristics

From November 2008 to May 2015, 275 patients were enrolled in the study. After screening, 19 patients were excluded because of refusal of treatment, resectable disease or other reasons. 3 patients missed follow-up within the first two treatment cycles. All patients were followed up with comprehensive information and the last date of follow-up was October 31, 2015. The baseline characteristics of the patients are shown in Table 1. The two groups were well balanced in respect to gender, age, site of location, anemia, histologic subtype, unresectable reason, and clinical stage.

**Table 1:** Baseline characteristics (*N* = 242)

Clinical features	EOX (group A; *N* = 112) No. of patients (%)	XELOX (group B; *N* = 130) No. of patients (%)	*P* value
Gender (N)			
Male	86/112 (76.8)	101/130 (77.7)	0.866
Female	26/112 (23.2)	29/130 (22.3)	
Age (median year, range)			
Median	56 18-75	62 31-78	
Range			
<65	80 (71.4)	84 (64.6)	0.32
Location (N,)			
Esophagogastric junction	25/112 (22.3)	43/130 (33.1)	0.086
Stomach	87/112 (77.7)	87/130 (66.9)	
Lauren type (N)			
Intestinal type	53/112 (47.3)	74/130 (56.9)	0.063
Diffuse type	46/112 (41.1)	35/130 (26.9)	
Mixed type	13/112 (11.6)	21/130 (16.2)	
CEA (N)			
Normal	80/112 (71.4)	96/130 (73.8)	0.782
Elevated	32/112 (28.6)	34/130 (26.2)	
Causes of unresection			
Exploration	4/112 (3.5)	7/130 (5.4)	0.759
T4b	47/112 (42.0)	56/130 (43.1)	
Bulky lymph nodes	61/112 (54.5)	67/130 (51.5)	
Clinical T stage			
cT3	2/112 (1.8)	6/130 (4.6)	0.386
cT4	110/112 (98.2)	124/130 (95.4)	
Clinical N stage			
cN1	31/112 (27.7)	34/130 (26.2)	0.273
cN2	41/112 (36.6)	60/130 (46.2)	
cN3	40/112 (35.7)	36/130 (27.6)	

EOX epirubicin, oxaliplatin and capecitabine; XELOX capecitabine and oxaliplatin;

### Response to the chemotherapy (Table 2)

After 4 cycles, there were 241 patients evaluable for response (one patient had acute stomach perforation after the first cycle of XELOX regimen and thus did not receive the evaluation). The result showed no difference in two groups. In group A, one patient had a complete response (CR), 36 had partial responses (PR), 56 had stable disease (SD), and 19 had progression of disease (PD). The response rate (RR) was 33.0% and disease control rate (DCR) was 83.0%. While in group B, RR and DCR were 33.8% and 88.4% respectively, which suggested no significant difference in response rates between the two groups.

**Table 2:** Response of preoperative chemotherapy and surgery resection rate in the two groups (*N* = 242)

	EOX (group A; *N* = 112) No. of patients (%)	XELOX(group B; *N* = 130) No. of patients (%)	*P* value
Response evaluation			
CR	1/112 (0.9)	2/130 (1.5)	0.378
PR	36/112 (32.1)	42/130 (32.3)	
SD	56/112 (50.0)	71/130 (54.6)	
PD	19/112 (17.0)	14/130 (10.8)	
Not assessable	0/112 (0)	1/130* (0.8)	
RR (CR plus PR)	37/112 (33.0)	44/130 (33.8)	0.997
DCR (CR plus RR plus SD)	93/112 (83.0)	115/130 (88.4)	0.305
Patients received surgery			
Radical surgery	63/112 (56.3)	81/130 (62.3)	0.408
Palliative surgery	19/112 (17.0)	9/130 (6.9)	
No surgery	30/112 (26.7)	40/130 (30.8)	

* One did not have response evaluation because of acute stomach perforation five days after the first cycle of chemotherapy. CR complete response PR partial response SD stable response PD progression of disease RR response rate DCR disease control rate

### Surgical findings and pathology staging (Table 3)

Ultimately, 63 patients in group A and 81 in group B achieved R0 resection. Nevertheless, the rate of resection with curative intent was similar in two groups (56.3% *vs*. 62.3%, *P* = 0.408). In patients who had radical operation, 44 cases in group A (44/63, 69.8%) had pathological responses and four of them (4/63, 6.3%) had complete pathological responses (pCR). While in group B, 47 patients (47/81, 58.0%) had pathological response and eight patients (9.9%) had pCR. Fifty-four (85.7%) patients in group A and 70 (86.4%) patients in group B had radical surgery with D2 lymphadenectomy. The median number of dissected lymph nodes (30 *vs*. 29) was close in both groups. Median positive lymph nodes were also no different between two groups (3 *vs*. 2) respectively. The median time from surgery to discharge both was 9d (range, 5-43d and 6-72d). Three cases in group A and one in group B had postoperative complications described as lung infection and pancreatic fistula after surgery.

**Table 3:** Surgical findings for the patients received radical surgery after chemotherapy (*N* = 144)

	EOX (group A; *N*=63) No. of patients (%)	XELOX (group B; *N*=81) No. of patients (%)	*P* value
Pathological response			
Responders	44/63 (69.8)	47/81 (58.0)	0.199
pCR	4/63 (6.3)	8/81 (9.9)	0.207
Patients received D2 lymphadenectomy	54/63 (85.7)	70/81 (86.4)	0.903
Median total nodes	30 (4-71)	29 (2-66)	0.754
Median positive nodes	3 (0-34)	2 (0-31)	0.421
Median time from surgery to discharge	9 (5-43)	9 (6-72)	0.752
Pathological T stage			0.260
ypT0	6/63 (9.5)	9/81 (11.1)	
ypT1	6/63 (9.5)	7/81 (8.6)	
ypT2	11/63 (17.5)	8/81 (9.9)	
ypT3	20/63 (31.7)	22/81 (27.2)	
ypT4a	18/63 (28.6)	33/81 (40.7)	
ypT4b	2/63 (3.2)	2/81 (2.5)	
Pathological N stage			0.905
ypN0	22/63 (34.9)	30/81 (37.1)	
ypN1	11/63 (17.5)	12/81 (14.8)	
ypN2	10/63 (15.9)	13/81 (16.0)	
ypN3a	15/63 (23.8)	17/81 (21.0)	
ypN3b	5/63 (7.9)	9/81 (11.1)	
Patients with T downstage	42/63 (66.6)	46/81 (56.8)	0.301
Patients with N downstage	32/63 (50.1)	42/81 (51.9)	0.689

### Survival

**Figure 1:**
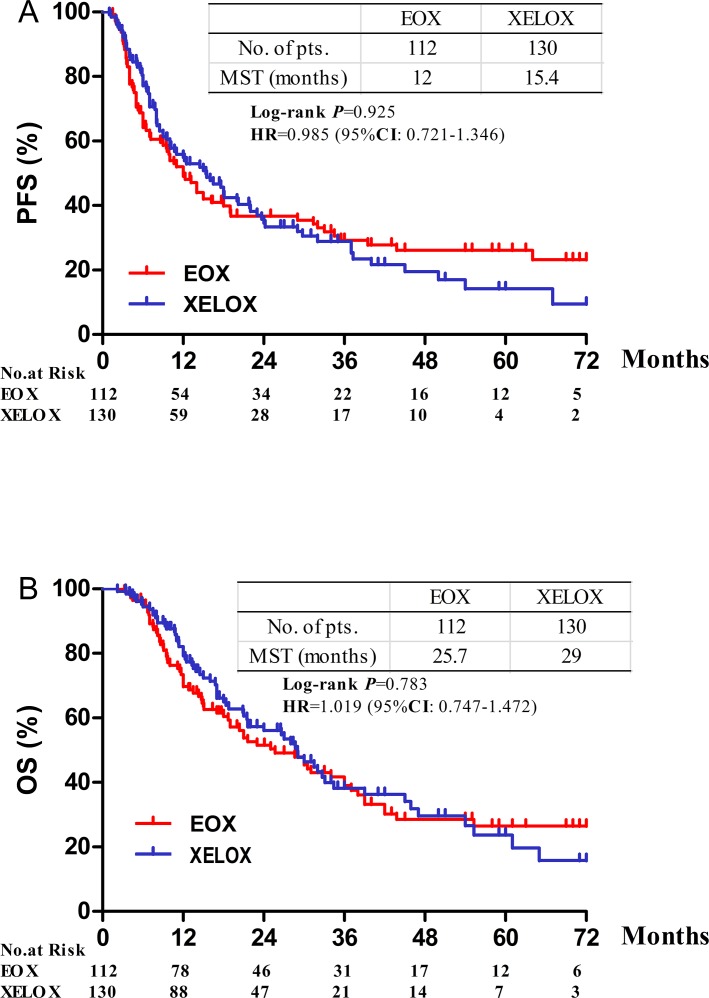
Progression-free and overall survival according to treatment **A.** Progrssion-free survival. **B.** Overall survival. HR = hazard ratio

After a median follow-up of 17.4 months (range 2.2-83.2 months), 163 patients (77 in group A and 86 in group B) had disease progression or relapsed, 106 patients (69 in group A and 67 in group B) died. The median OS was 25.7 months (95% CI 17.2-34.1) in group A and 29.0 months (95% CI 22.1-35.8) in group B (HR 1.019; 95%CI 0.747-1.472, *P* = 0.783). The median PFS was 12.0 months (95% CI 8.7-15.2) and 15.4 months (95% CI 10.2-20.5) for group A and B, respectively (HR 0.985; 95% CI 0.721-1.326, *P* = 0.925, Figure 1).

**Figure 2:**
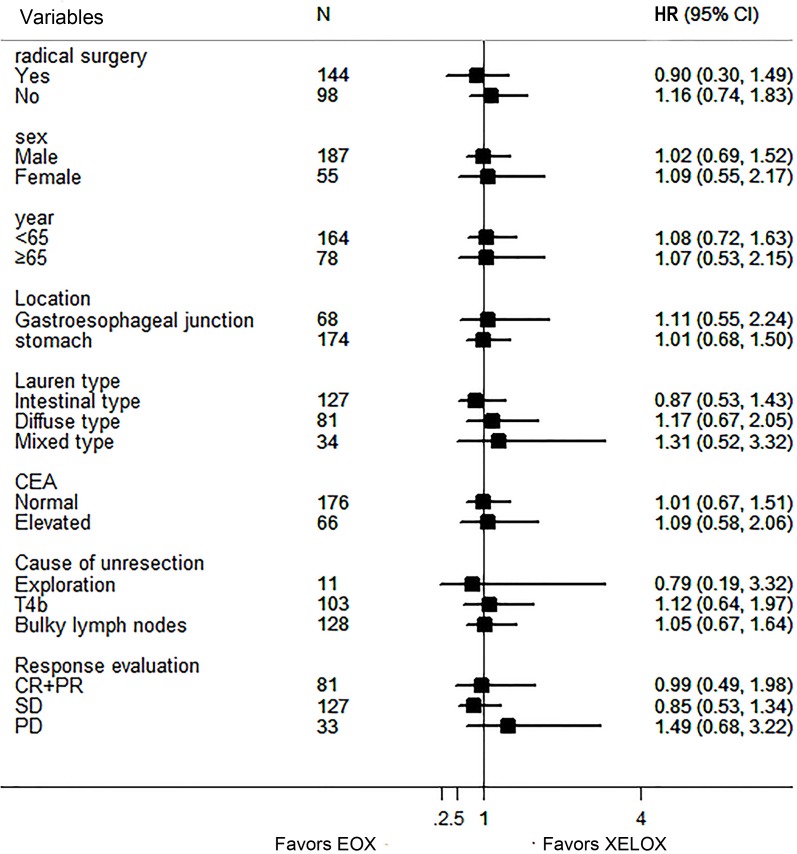
Forest plot of the treatment effect on overall survival in subgroup analysis

**Figure 3:**
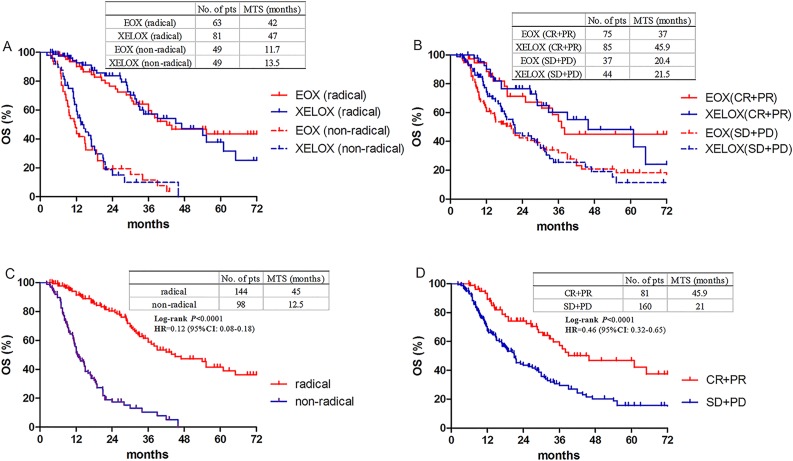
Overall survival according to resection and response **A.** Overall survival in patients with or without radical resection between EOX and XELOX groups. **B.** Overall survival in responders or non-responders between EOX and XELOX groups. **C.** Overall survival in patients with or without radical resection. **D.** Overall survival in responders or non-responders. Responders mean CR and PR, non-responders mean SD and PD.

Subgroup analysis showed no statistical benefit favored any regimen in any variables (Figure 2). Patients who underwent a radical resection had similar overall survival between two groups, as well as those who had tumor response (Figure 3A and 3B). Patients who had undergone a radical resection had a significant longer median OS of 45 months (95% CI 32.4-57.4 months) than patients without radical resection (12.5months, 95% CI 9.9-15.0 months, HR 0.12; 95%CI 0.08-0.18, *P* < 0.0001, Figure 3C). Resection followed response to chemotherapy, and tumor response itself had a major influence on the OS (HR 0.46; 95%CI 0.32-0.65, *P* < 0.0001, Figure 3D).

### Toxicity (Table 4)

Overall, the toxicity observed was mostly mild in both groups, and no deaths were attributable to chemotherapy or surgery. The most common adverse events were gastrointestinal issues and leukocytopenia. Group A experienced more serious leukocytopenia (22.3% *vs*. 10.0%, *P* = 0.014), neutropenia (23.2% *vs*. 11.5%, *P* = 0.025), nausea (11.6% *vs*. 3.8, *P* = 0.041) and vomiting (10.7% *vs*. 2.3, *P* = 0.015).

**Table 4:** Grade 3/4 events in the whole population (*N* = 242)

Toxicities	EOX (group A; *N* = 112) No. of patients (%)	XELOX (group B; *N* = 130) No. of patients (%)	*P* value
*Hematological*			
Leukocytopenia	25/112 (22.3)	13/130 (10.0)	**0.014**
Neutropenia	26/112 (23.2)	15/130 (11.5)	**0.025**
Febrile neutropenia	4/112 (3.5)	1/130 (0.8)	0.282
Thrombocytopenia	11/112 (9.8)	20/130 (15.4)	0.272
Anemia	9/112 (8.0)	14/130 (10.1)	0.614
*Non-hematological*			
Nausea	13/112 (11.6)	5/130 (3.8)	**0.041**
Vomiting	12/112 (10.7)	3/130 (2.3)	**0.015**
Diarrhea	4/112 (3.5)	5/130 (3.8)	0.820
Hand-foot skin reaction	3/112 (2.7)	4/130 (3.1)	0.841
Hepatic dysfunction	4/112 (3.5)	6/130 (4.6)	0.933
Neuropathy	7/112 (6.2)	15/130 (11.5)	0.229
Mucositis	4/112 (3.6)	6/130 (4.6)	0.934
Cardiac side effect	1/112 (0.9)	0/130 (0)	0.940

## DISCUSSION

Optimal regimen as preoperative chemotherapy in the locally advanced gastric cancer is still a matter of debate. This study is the first to evaluate the feasibility and the potential benefit of preoperative chemotherapy with or without the addition of epirubicin to capecitabine and oxaliplatin in initially unresectable Chinese gastric cancer patients. We confirmed that even additional epirubicin was added, EOX regimen could not show more advantages compared to XELOX regimen in aspects of response rate, conversion rate from unresectable to resectable and survival. The result was similar with other studies in assessing difference between two-drug and three-drug regimens as preoperative or first-line chemotherapy treatment [16, 17]. Lorenzen S demonstrated [16] that neoadjuvant FLOT (infusional 5-FU, leucovorin, oxaliplatin and docetaxel) offered an acceptable chance of curative surgery compared with FLO (infusional 5-FU, leucovorin, and oxaliplatin) in elderly patients with locally advanced gastroesophageal cancer, while a French Intergroup Study [17] even showed that doublet FOLFIRI (Fluorouracil, Leucovorin, and Irinotecan) as first-line treatment for advanced gastric cancer had significantly better TTF (Time to failure) than triplet ECX (Epirubicin, Cisplatin and Capecitabine).

In addition, it was apparently known that more aggressive therapy containing triplet combinations had more adverse effects. Our data indicated that preoperative chemotherapy related toxicity events tended to be more frequent in EOX group than XELOX group, especially in leucopenia, fatigue and vomiting. Furthermore, only 44.4% of patients who had radical surgery in group A (28/63) completed all eight cycles of treatment; 20 patients (31.7%) received XELOX regimen as postoperative chemotherapy predominantly owing to postoperative complications, while 58 of 81 patients (71.6%) who underwent radical resection in Group B completed all eight cycles of XELOX regimen. The accomplished ratio in group A was similar with MAGIC study which was 42% as reported. The tolerance of postoperative chemotherapy was more acceptable in the epirubicin free regimen. Epirubicin as well as methotrexate and mitomycin C had been used as component of chemotherapy regimen in gastric cancer since the late 1980s [18–20], however these old agents have been less applied nowadays as their low activities and high toxicities, especially in Eastern countries. Meanwhile, the dose of capecitabine in XELOX regimen is higher than EOX regimen which could partially explain the identical response between doublet and triplet regimens. As newer chemotherapy agents have become more and more available such as docetaxel, paclitaxel and irinotecan which are demonstrated promising efficacy and manageable toxicity [17, 21–23], new triplet regimens including more powerful agents should be considered.

In this study, we also confirmed that patients could have survival benefit from radical resection after response to the preoperative chemotherapy [7, 24–25]. Median OS could be prolonged from 12.5m to 45m when conversion chemotherapy and radical surgery were sequentially accomplished. There were no difference regarding the R0 resection rate compared our study with others evaluating the efficacy and feasibility of different preoperative chemotherapy regimens in patients with initially unresectable locally advanced gastric cancer [7, 24, 25], which were from 48.1% to 63%. A few of patients who got stable disease evaluated by CT scan were diagnosed as being resectable when assessed by a multidisciplinary team, which explained higher R0 resection ratio than response rate. Meanwhile, the radiological response could predict that patients who obtained CR or PR would have better survival than these achieved SD or PD (45.9m *vs*. 21.0m), which indicated that response to preoperative chemotherapy may predict survival before curative resection of gastric cancer.

Since it is a prospective observational study, there are some limitations that cannot be avoided, such as non-randomized, unblinded setting which might have a great chance producing selection bias. However, figures in Table 1 showed that basic characteristics were well balanced between the two groups.

In conclusion, our study suggested that unresectable locally advanced gastric cancer patients can benefit equally from XELOX and EOX regimen. As the more aggressive treatment including three chemotherapeutic drugs would produce more toxicity effect, mild doublet treatment might be a better choice. Further randomized trials focused on certain type of regimen will help us to determine which strategy is the best for these patients.

## SUPPLEMENTARY MATERIAL TABLE


