# Supplementary material for: Efficacy of preoperative chemotherapy regimens in patients with initially unresectable locally advanced gastric adenocarcinoma: capecitabine and oxaliplatin (XELOX) or with epirubicin (EOX)
Source: Oncotarget. 2016 Sep 1;7(46):76298–307. doi: 10.18632/oncotarget.11818 (PMC5342815; doi:10.18632/oncotarget.11818)
Supplement: Supplementary file 1 [file oncotarget-07-76298-s001.pdf]

Efficacy of preoperative chemotherapy regimens in patients with initially unresectable locally advanced gastric adenocarcinoma: capecitabine and oxaliplatin (XELOX) or with epirubicin (EOX)

Supplementary Material

Supplement Table 1. Characteristics of patients received radical srugery (N=144).

Clinical features	EOX (group A; N=63)	XELOX (group B; N=81)
Gender (N)		
Male	48/86	62/101
Female	15/26	19/29
Location (N,)		
Gastroesophageal junction	12/25	28/43
Stomach	51/87	53/87
Lauren type (N)		
Intestinal type	31/53	49/74
Diffuse type	27/46	18/35
Mixed type	5/13	14/21
CEA (N)		
Normal	44/80	64/96
Elevated	19/32	17/34
Causes of unresection		
Exploration	1/4	4/7
T4b	28/47	40/56
Bulky lymph nodes	34/61	37/67
Clinical T stage		
cT3	2/2	3/6
cT4	61/110	78/124
Clinical N stage		
cN1	19/31	26/34
cN2	23/41	40/60
cN3	21/40	15/36
Response evaluation		
CR	1/1	2/2
PR	30/36	33/42
SD	32/56	46/71
PD	0/19	0/14
